# Oral Health Coaches at Well-Baby Clinics to Promote Oral Health in Preschool Children From the First Erupted Tooth: Protocol for a Multisite, Pragmatic Randomized Controlled Trial

**DOI:** 10.2196/39683

**Published:** 2022-08-31

**Authors:** Peggy C J M van Spreuwel, Katarina Jerković-Ćosić, Cor van Loveren, Geert J M G van der Heijden

**Affiliations:** 1 Research Group Innovation in Preventive Care Hogeschool Utrecht University of Applied Science Utrecht Netherlands; 2 Oral Public Health Department Academic Centre for Dentistry Amsterdam University of Amsterdam and Vrije Universiteit Amsterdam Netherlands; 3 Academic Centre for Dentistry Amsterdam University of Amsterdam and Vrije Universiteit Amsterdam Netherlands

**Keywords:** randomized clinical trial, dental caries, early childhood caries, oral health promotion, behavior change, motivational interviewing, dental public health, child health care, health inequality, prevention

## Abstract

**Background:**

Early childhood caries is considered one of the most prevalent diseases in childhood, affecting almost half of preschool-age children globally. In the Netherlands, approximately one-third of children aged 5 years already have dental caries, and dental care providers experience problems reaching out to these children.

**Objective:**

Within the proposed trial, we aim to test the hypothesis that, compared to children who receive usual care, children who receive the Toddler Oral Health Intervention as add-on care will have a reduced cumulative caries incidence and caries incidence density at the age of 48 months.

**Methods:**

This pragmatic, 2-arm, individually randomized controlled trial is being conducted in the Netherlands and has been approved by the Medical Ethics Research Board of University Medical Center Utrecht. Parents with children aged 6 to 12 months attending 1 of the 9 selected well-baby clinics are invited to participate. Only healthy children (ie, not requiring any form of specialized health care) with parents that have sufficient command of the Dutch language and have no plans to move outside the well-baby clinic region are eligible. Both groups receive conventional oral health education in well-baby clinics during regular well-baby clinic visits between the ages of 6 to 48 months. After concealed random allocation of interventions, the intervention group also receives the Toddler Oral Health Intervention from an oral health coach. The Toddler Oral Health Intervention combines behavioral interventions of proven effectiveness in caries prevention. Data are collected at baseline, at 24 months, and at 48 months. The primary study endpoint is cumulative caries incidence for children aged 48 months, and will be analyzed according to the intention-to-treat principle. For children aged 48 months, the balance between costs and effects of the Toddler Oral Health Intervention will be evaluated, and for children aged 24 months, the effects of the Toddler Oral Health Intervention on behavioral determinants, alongside cumulative caries incidence, will be compared.

**Results:**

The first parent-child dyads were enrolled in June 2017, and recruitment was finished in June 2019. We enrolled 402 parent-child dyads.

**Conclusions:**

All follow-up interventions and data collection will be completed by the end of 2022, and the trial results are expected soon thereafter. Results will be shared at international conferences and via peer-reviewed publication.

**Trial Registration:**

Netherlands Trial Register NL8737; https://trialsearch.who.int/Trial2.aspx?TrialID=NL8737

**International Registered Report Identifier (IRRID):**

DERR1-10.2196/39683

## Introduction

Dental caries (or tooth decay) represents a major public health problem affecting almost half of preschool-age children worldwide [1]. Early childhood caries (ECC) may have severe consequences on daily functioning and socialization in young children [2,3]. While dental caries is highly preventable with twice-daily toothbrushing using fluoridated toothpaste and limited sugar intake between main meals, compliance with these measures still requires much perseverance and endurance from parents. Noncompliance is often related to a lack of awareness among parents about their role in preventing ECC, a persistent belief that primary teeth are not important for oral health in later life, power struggles with the child, and tiredness of parents and their children [4-6]. As with many behavior-related diseases, dental caries shows marked socioeconomic disparities in all age groups [7,8].

Life-course epidemiology has highlighted that early childhood is critical for developing good oral health [9,10]. Positive oral hygiene behaviors and skills should be introduced early in the child’s life, and parents need to acquire the knowledge to develop proper oral hygiene behaviors and skills [10]. This knowledge about the importance of caries prevention in early childhood contributes to the recommendation to have an early dental visit before the age of 1 year [11]. However, while in the Netherlands parents’ compulsory health care insurance fully covers oral health care for their children up to the age of 18 years, oral health care professionals experience difficulties reaching children below 5 years of age, particularly those with lower socioeconomic backgrounds. As a result, less than half of Dutch children have visited an oral health professional at the age of 4 years, and dentin caries is present in about one-third of Dutch children aged 5 years [12,13]. At the same time, more than 90% of all newborns, including children from low socioeconomic backgrounds, visit well-baby clinics (WBCs) for preventive health care and vaccinations at regular intervals up to the age of 4 years [14]. Therefore, WBCs provide a unique window of opportunity for early oral health promotion.

Nevertheless, due to lack of time, medical and nursing staff at WBCs pay little attention to oral health promotion and timely referral to oral health professionals, prioritizing other child health issues over oral health promotion [15]. The second problem is that even when children visit an oral health professional on time, there is a lack of evidence-based interventions to provide appropriate preventive oral care starting from the first tooth [16]. Therefore, there is a double challenge in preventing poor oral health in early childhood. First, to reach young children with their parents in a timely fashion, and second, to develop and provide effective preventive oral health care and oral health promotion to address behavioral risk factors for poor oral health.

The Toddler Oral Health Intervention (TOHI; *Gezonde Peutermonden* in Dutch) has been developed to address these challenges by providing proven, effective early interventions. Firstly, in the TOHI, an oral health coach (OHC) is assigned by a dental clinic to participating WBCs in the same neighborhood. For children aged 6 to 12 months up to 48 months attending WBCs, the TOHI combines the regular WBC appointments with an appointment with the OHC. In doing so, the TOHI follows the example of the nationwide Scottish Childsmile program [17,18]. This program has successfully reached very young children and their parents through WBCs. Secondly, the OHC works according to the Non-Operative Caries Treatment And Prevention (NOCTP) approach, which has been reported to result in a 40% reduction in caries incidence in Dutch schoolchildren [19]. Thirdly, the OHC will use motivational interviewing (MI). While MI was initially developed as a behavioral technique for treating substance abuse [20], it has been shown to be effective in contributing to the prevention of ECC. MI has led to a reduction in caries incidence ranging from 16% to 26% and a 30% to 55% reduction in the number of decayed teeth [21-24]. Finally, the Health Action Process Approach (HAPA) [25], a theoretical framework to explain, predict, and modify health behaviors, provides tools to focus on the underlying determinants of behavior. By understanding the stages of behavioral change and focusing the intervention on the associated determinants, the impact of NOCTP and MI can be increased. The importance of determinants that underpin motivational and self-regulatory processes and help translate intention into behavior in oral health care, such as attitudes, self-efficacy, planning, and action control, has been demonstrated in previous research [26-29]. As the TOHI combines different existing components of health care and works on different organizational levels, it can be considered a complex intervention following the definition of the Medical Research Council (MRC) [30].

This paper describes a detailed research protocol to assess the effectiveness of the TOHI as an addition to usual WBC care, compared to usual WBC care only, in children aged up to 48 months. We hypothesize that the TOHI as an addition to usual care will reduce cumulative caries incidence at the age of 48 months (ie, the primary outcome measure), the sum of the number of decayed, missing, and filled surfaces and teeth (dmfs/dmft), the caries incidence density (person-time at risk to the first cavity), the presence of dental plaque, and the consequences of untreated caries (ie, secondary oral health outcome measures). We will study and report on (1) the oral health outcomes, (2) the behavioral outcomes, and (3) the balance between costs and effects of the TOHI.

## Methods

### Trial Design and Setting

**Figure 1:**
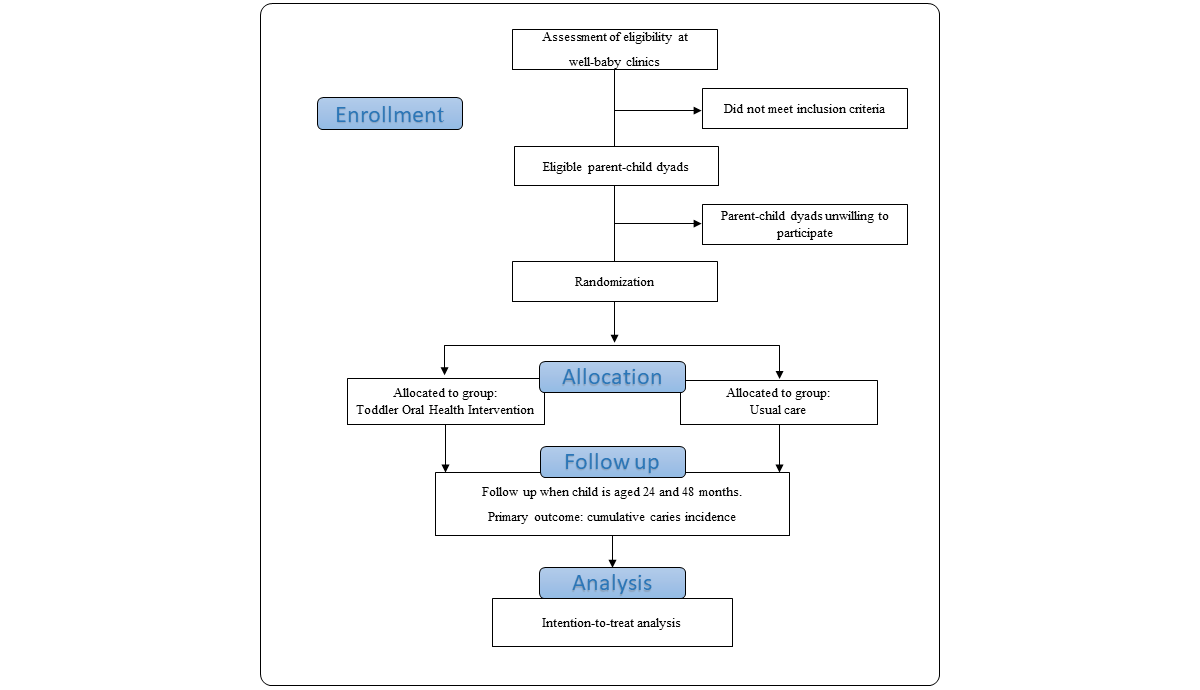
Outline of the randomized controlled trial.

This protocol describes a pragmatic, 2-arm, individually randomized controlled trial (RCT) conducted in the Netherlands. The study population was recruited at 9 WBCs in urban and suburban regions with a population of predominantly low to middle socioeconomic position (SEP). Figure 1 shows a flow chart of the study’s overall design. This paper has been prepared following the Standard Protocol Items: Recommendations for Interventional Trials (SPIRIT) reporting guidelines [31] and the Template for Intervention Description and Replication (TIDieR) [32].

### Ethical Considerations

Prior to study initiation, the research protocol was submitted to the Medical Ethics Committee of the University Medical Centre Utrecht for review and approval. The committee provided ethics clearance (NL60021.041.17; file number 17-133/D) and stated that the research complied with the applicable rules and requirements of the Medical Research Involving Human Subjects Act (Dutch abbreviation: WMO) and with the ethics code for the conduct of research as set out in the national Code of Conduct for Scientific Integrity in the Netherlands [33]. Participation was voluntary, and participants could withdraw at any moment with no consequences. Participants signed an informed consent form before enrollment. The trial is registered in the Netherlands Trial Register (NL8737).

### Participants and Eligibility Criteria

This study includes parent-child dyads with children aged 6 to 12 months. The study excludes dyads with (1) children in need of specialized health care for a physical, mental, or medical condition; (2) parents with insufficient knowledge of Dutch; and (3) parents who expected to move outside the region of the WBC within the duration of the study. In the case of twins, only 1 child is enrolled in the study.

### Sample Size Calculation

Previous studies show that the average number of caries lesions in Dutch children at the age of 5 years is 4.5 (SD 4.5) [12]. This information was used for study size estimation using G*power 3 [34]. This showed that the intervention group needed at least 149 participants to demonstrate a reduction in cumulative caries incidence of at least 30% with a statistically significant Mann-Whitney *U* test allowing for statistical power (1-β) of .8 and a 2-sided α of .05 as a threshold for statistical significance. With an anticipated 30% loss to follow up, the aim was to include at least 200 participants per group.

### Intervention Randomization

With a 1:1 ratio, participating parent-child dyads were individually allocated to the intervention or control group. This allocation of the TOHI as an add-on to usual care was stratified for each WBC (9 in total) and educational level of the mother (classed as low, medium, or high) as an indicator of SEP. A computerized random sequence generator [35] with a block size of 4 to ensure equally filled strata in each group was used to allocate the intervention status to predefined unique ID numbers. Accordingly, an intervention allocation file was prepared, including intervention codes with blocked series of ranked unique participant IDs for the strata.

### Informed Consent and Concealment of Intervention Allocation

Research nurses who were not involved in the intervention allocation determined the eligibility of parent-child dyads attending the selected WBCs. First, all eligible dyads received verbal and written information about what participation in the study entailed. Then, the parents or persons with parental legal authority signed the informed consent form to confirm voluntary participation. Upon receipt of this form, the principal investigator assigned a unique patient ID based on the WBC and education level of the mother. The corresponding intervention status was retrieved from the prepared allocation files of an independent data administrator. Next, the principal investigator contacted the parents to inform them about the allocated intervention and to ensure that they understood all the information about their participation.

### Blinding to the Nature of the Intervention

The research staff involved in clinical assessments are kept blinded to the nature of the allocated treatment. Due to the nature of the intervention, blinding of participating parent-child dyads has not been possible. To maintain the blinding of outcome assessors, parents are repeatedly instructed not to talk about the intervention they received with the assessors. The success of the blinding of the outcome assessors is tested by asking them the following question: “To which group is this child assigned according to your opinion?” Possible answers are as follows: “Control group: I know for sure,” “Intervention group: I know for sure,” “Control group: I think so,” “Intervention group: I think so,” and “I do not know” [36,37].

### Intervention Development

The MRC guidance on developing and evaluating complex clinical health care interventions was utilized in development and evaluation of the TOHI [30]. Prior to this study, the feasibility of the TOHI was investigated in a small-scale feasibility study and evaluated through semistructured interviews with stakeholders, including oral health professionals, OHCs, youth health care professionals, and parents. The resulting findings were instrumental in the study design and were used to refine the TOHI and adapt it to participating WBCs in the trial. Textbox 1 presents the conceptual framework for the TOHI. A full description of the TOHI, following TIDieR reporting guidelines [32], is available in Multimedia Appendix 1. The different components of the TOHI are described below.

**Conceptual framework of the Toddler Oral Health Intervention.**

Components of the Toddler Oral Health Intervention

- Using well-baby clinics to reach parents with newborn children
- Using the Non-Operative Caries Treatment And Prevention Method for individual risk assessment and preventive oral care
- Using motivational interviewing as the main tool to elicit internal motivation for desired behavior
- Using the Health Action Process Approach behavioral theory to guide the intervention based on underlying determinants of behavior

Expected mediators and secondary outcomes

- Increased self-efficacy
- Increased outcome expectancies
- Increased risk perception
- Intention forming for favorable oral health behavior; oral health coaches will explore barriers to and facilitators of favorable oral health behavior and focus on action planning and coping planning for favorable oral health behavior
- Action and maintenance of favorable behavior; if necessary, oral health coaches will work on recovery self-efficacy

Oral health behaviors

- Twice-daily toothbrushing with fluoride toothpaste
- Improved dietary habits with respect to oral health (eg, limiting sugar intake and the use of bottle-feeding, and drinking water more often)

Primary oral health outcome

- Reduced cumulative caries incidence

### Interventions

In both the intervention and the control group, all dyads received or are receiving preventive child health care at the WBCs, which are regulated under the Public Health Act 2008. When a baby is registered in a municipality in the Netherlands, parents receive an invitation from the nearest WBC for a series of at least 13 appointments between birth and the age of 4 years. The goals of this preventive health care program are to monitor growth and development, detect health and social problems (or risk factors) at an early stage, screen for metabolic conditions and hearing in the newborn, deliver the national vaccination program, and provide advice and information on health [14]. According to the Public Health Act, this advice includes brief oral health promotion, such as the use of fluoridated toothpaste (at 500 ppm-750 ppm) and the advice to visit an oral health professional within the first year of life (Dutch Public Health Act, Article 6, paragraph 1, Dutch Public Health Decree). Dyads enrolled in the intervention group receive the TOHI in addition to this preventive health care program. Dyads enrolled in the control group receive only the usual care that all children receive at all WBCs in the Netherlands.

The TOHI builds on four components: (1) visiting an OHC at a WBC, (2) training OHCs to work accord to the NOCTP method, (3) using MI, and (4) using HAPA. The general understanding underlying the TOHI is that caries is a localized process and to a large extent can be prevented by brushing the teeth with fluoride toothpaste and lowering the frequency of sugar intake. With this understanding in mind, the OHC aims to create awareness among parents that through establishing adequate oral health behavior, they can effectively contribute to preventing caries in their children. The standardized protocol of the NOCTP method contributes to a uniform and transparent approach for OHCs and parents [19]. The method assigns 1 point based on parental cooperation and motivation, measured by the child’s oral hygiene and whether they follow the key oral health messages of the Ivory Cross national guidelines [38], and 1 point for active or incipient caries or lesions (Table 1). MI is used in the TOHI to elicit parents’ internal motivation and explore barriers and facilitators for desired oral health behavior. Finally, HAPA is a hybrid behavioral model that explicitly outlines 2 different phases of health behavior change: a motivational, intention-forming phase and a volitional phase in which translation of intention into action takes place [25]. Guided by HAPA, the OHCs focus on motivational and self-regulatory processes, such as self-efficacy, planning, and action control, which have been shown to be mediators in translating intentions into behaviors [29]. The OHC combines the NOCTP protocol with an intervention guided by HAPA and MI skills to determine which aspects of behavior need to be addressed for a tailored intervention.

**Table 1:** Criteria for caries risk assessment, recall interval, and intervention.

Caries risk score	Risk	Intervention	Interval
0	Low	Periodic oral examination	Combined with scheduled well-baby clinic appointment within 6 to 12 months
1 (without active caries lesions)	Average	Shortened interval for periodic oral examination and exploration of barriers to and facilitators of self-care agreements	Combined with scheduled well-baby clinic appointment within 3 to 6 months or extra appointment when indicated
1 or 2 (with active caries lesions)	High	Shortened interval for periodic oral examination and exploration of barriers to and facilitators of self-care agreements; if necessary, referral to a dental office for fluoridation or treatment of caries lesions or incipient caries lesions	Combined with scheduled well-baby clinic appointment within 1 to 3 months or extra appointment when indicated

### Procedures of the TOHI

Face-to-face, 10-to-20-minute appointments with an OHC were combined with the child health care appointments at the WBC at the ages of 6 or 8, 11, 15, 18, 24, 36, and 42 months. Every appointment with the OHC started with an assessment of the child’s caries risk, following the NOCTP protocol. Subsequently, the OHC adjusted the oral health promotion to this risk by targeting the mediating determinants according to the HAPA, depending on the parents’ health behavior phase. When the intention was formed, self-care agreements were made in the volitional phase that focused on planning and action control to translate intentions into behaviors. Additionally, ambivalent feelings and barriers or facilitators to self-care agreements were explored using MI to stimulate intrinsic motivation and reinforce parents in existing positive behavior. According to the NOCTP protocol, when an increased caries risk was detected, the OHC could decide to schedule an additional appointment. If incipient caries lesions were detected, parents were educated on how to clean the caries lesion, and the use of fluoride toothpaste was again emphasized. When dental treatment for caries was deemed necessary, the OHC referred the participants to a dental clinic.

### Materials Used for the TOHI

A penlight and disposable materials (eg, cotton rolls to dry elements, dental mirrors, and wooden toothpicks to detect dental plaque on elements) were used for oral assessments by the OHC. In addition, the OHC used predefined paper patient records to ensure efficient and uniform reporting. These paper records contained age-related oral health topics to discuss, a caries risk assessment according to the NOCTP protocol, and an assessment of the parents’ oral health behavioral stage. The final caries risk and behavioral stage guided the OHC to follow-up actions, such as which mediating determinants from the HAPA should be addressed with the intervention (Multimedia Appendix 2). Parents in the intervention group received a specially developed TOHI booklet at baseline. This booklet was developed for 2 reasons. First, it contained oral health reports for each appointment with the OHC, which effectively communicated the child’s caries risk to the parents. These reports also covered agreed self-care actions to reduce caries risk. Together with the parents, the OHC examined and recorded the barriers to self-care actions and the mediating determinants of self-efficacy, action planning, and coping planning in these reports (Multimedia Appendix 3). Finally, the booklet covered essential oral health information and provided tips and tricks that the OHC could refer to.

### Oral Health Coaches and Training

Eligible OHCs for this study had a background in dentistry (they were dentists, dental hygienists, or dental assistants). They were prevention-minded, trained (or willing to be trained) in the NOCTP method, had good social communication skills, and had experience in pediatric dentistry. In addition, all OHCs received training in the TOHI delivered by a certified MI trainer and the principal investigator, who are both experienced in training oral health professionals and students in NOCTP, HAPA, and MI. A pediatric dietitian provided extra training on infant and toddler nutrition related to oral health. The first training session took place before the study started. Subsequently, training continued every 3 to 4 months for the duration of the study. During this training, the focus was on recognizing stages of health behavior, using HAPA, and developing advanced MI skills, such as recognizing and managing behavioral resistance and detecting ambivalence. Learning these skills takes time and is an ongoing process. Therefore, the training consisted of presenting practical examples of (successfully resolved) complex cases provided by the OHCs, role-playing, and peer feedback. In addition, the MI trainer provided individual feedback via self-recorded audio fragments that were uploaded by the OHCs. These audio fragments were assessed biannually with the Motivational Interviewing Treatment Integrity (MITI) [39] system by an independent MI expert. The results of the intervention fidelity will be analyzed and prepared for an original process evaluation article alongside the article reporting the results of the effectiveness study.

### Design of Data Collection

The outcome measures and additional data were collected with online questionnaires at baseline and with online questionnaires and clinical examinations when the children reached the ages of 24 and 48 months. Table 2 provides an overview of measurements and timepoints.

**Table 2:** Measurements and timepoints.

Timepoint		Study period			
		–T1^a^	T0^b^	T1^c^	T2^d^
**Enrollment**					
	Eligibility screening	✓^e^			
	Informed consent	✓			
	Allocation	✓			
**Interventions**					
	Toddler Oral Health Intervention performed at ages 6-8, 11, 14-15, 18, 24, 30, and 36 months		✓	✓	✓
	Care as usual		✓	✓	✓
**Primary outcome**					
	Clinical assessment of cumulative caries incidence with Merged International Caries Detection and Assessment System [40]				✓
**Secondary outcomes**					
	Clinical assessment of average number of decayed, missing, and filled surfaces and teeth with Merged International Caries Detection and Assessment System			✓	✓
	Clinical assessment of person time at risk for first cavity with Merged International Caries Detection and Assessment System and incidence density			✓	✓
	Clinical assessment of consequences of untreated dental caries with the PUFA (visible pulpal involvement, ulceration caused by dislocated tooth fragments, fistula, and abscess) index			✓	✓
	Clinical assessment of presence of dental plaque with Simplified Oral Hygiene Index, 6-surface method [41]			✓	✓
	Online questionnaire assessment of oral health behavior with self-developed items		✓	✓	✓
	Online questionnaire assessment of psychosocial constructs of the Health Action Process Approach items (action self-efficacy, risk perception, outcome expectancies, intention, action planning, coping planning, and action control), adapted from Gholami and Schwarzer (2014) [42]		✓	✓	✓
	Online questionnaire assessment of oral health–related quality of life with the Early Childhood Oral Health Related Quality of Life [43] scale			✓	✓
	Online questionnaire assessment of child cooperativeness at oral examinations with the Frankl behavior scale [44]			✓	✓
**Compliance, fidelity, and cost outcome measures**					
	Total care delivered by oral health coach, assessed by frequency and time in minutes as reported by the oral health coach			✓	✓
	Reasons for and number of visits to a dental clinic (other than Toddler Oral Health Intervention), assessed by parent interviews				✓
	Oral health coach mastery of motivational interviewing competencies, assessed by applying Motivational Interviewing Treatment Integrity codes to audio recordings [39]		✓	✓	✓

^a^–T1: screening and allocation.

^b^T0: baseline.

^c^T1: follow-up at age 24 months.

^d^T2: follow-up at age 48 months.

^e^✓: measurement or action performed at this time point.

### Training of Outcome Assessors

The clinical outcome measures were collected by trained and calibrated dentists or dental hygienists in an assessor-blinded manner. The assessors were experienced in the use of the International Caries Detection and Assessment System (ICDAS) codes [40] for teaching or research purposes. Therefore, the training consisted of the online International Caries Classification and Management System (ICCMS) core e-learning [45] system and an online interrater reliability test developed for this study. This test presented 20 expertly verified high-quality pictures with different merged ICDAS scores in random order. If necessary for calibration, additional norming meetings were organized to maintain the performance of the assessors for the duration of the study.

### Outcome Assessments

**Figure 2:**
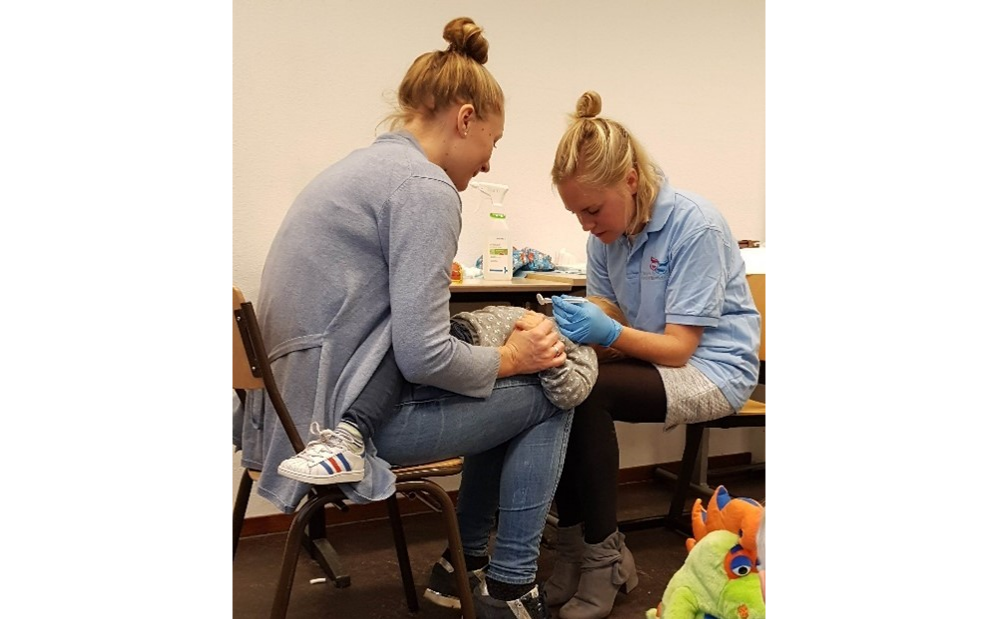
Knee-to-knee position for oral examination.

At 24 and 48 months, a knee-to-knee examination (Figure 2) is completed with the assistance of the parents or caregivers. The selected tooth surfaces are scanned from the incisal to the gingival edge with a toothpick to determine the presence of dental plaque. Next, the child’s teeth are cleaned and dried with cotton rolls or gauze to enable an adequate view of the tooth surfaces and systematic examination of each tooth for the presence of caries or incipient caries. Findings are recorded in an online case record form developed for this study by an assistant. If no assistant is available, an audio recording is used so that the assessor can work efficiently and speak their findings aloud, preventing recall bias in later reporting. In cases with findings such as dentine caries, caries-related inflammation, and eruption failure of the primary teeth, parents receive a letter with the advice to visit a dentist and the reasons for doing so. If necessary, the OHC provides a list of nearby dentists. After dental treatment, children in the intervention group return for follow up with the OHC.

### Primary Outcome

The cumulative caries incidence at the age of 48 months will be assessed by merged ICDAS [40] scores. These merged scores differentiate teeth as being sound or having initial, moderate, or severe caries.

### Secondary Outcomes

The average dmfs/dmft number and the caries incidence density (person-time at risk to the first cavity) will be calculated from the merged ICDAS scores. Consequences of untreated dental caries will be measured using the PUFA (visible pulpal involvement, ulceration caused by dislocated tooth fragments, fistula, and abscess) Index [46]. The presence of dental plaque will be determined with the 6-surface method of the Simplified Oral Hygiene Index [41]. Child cooperativeness during the outcome assessments will be documented by clinical assessors using the modified Frankl behavior scale [44].

Behavioral change over time will be assessed based on self-reported oral health behavior and psychosocial constructs from the HAPA, measured with online questionnaires at baseline, age 24 months, and age 48 months. The early childhood oral health–related quality of life will be measured using the Early Childhood Oral Health Impact Scale [43].

### Demographics and Patient Characteristics

The demographic characteristics include the children’s age, sex, family composition, mother’s highest educational attainment according to the International Standard Classification of Education [47], and health literacy measured with the 16-item European Health Literacy Survey Questionnaire (HLS-EU-Q16) [48].

### Design of Data Analyses

All analyses of the effectiveness of the TOHI will make use of the intention-to-treat principle. To prevent biased principal data analysis, concealment will be maintained during data analysis. The blinding of the study comparison will be broken only after the completion of data analysis for the evaluation of outcomes for children at the age of 48 months. After data cleaning and prior to data analysis, the amount and patterns of missing data will be explored. Sensitivity analysis will be used to explore the impact of missing data. The 3 following scenarios will be assumed: stability over time (last value carried forward or backwards to replace missing value), best case (largest positive change score observed to replace missing value), and worst case (largest negative change score observed to replace missing value).

The intervention effects for the cumulative caries incidence at 48 months and the average dmfs/dmft will be compared as the risk difference (additive scale), numbers needed to treat, and the relative risk (multiplicative scale), all with the 95% CI. Effects for subgroups (ie, WBC, education level of the mother, and health literacy) will be evaluated with interaction terms in a multivariate regression analysis. To determine incidence density and person-time at risk, the median and mean between-group differences in caries incidence (or the person-time to first caries lesion) will be based on a Kaplan-Meier analysis and incidence differences and ratios. In addition, when applicable, Cox proportional hazard regression analysis will be applied to explore relations with independent variables. Between-group differences at the age of 24 months will be assessed for the secondary outcomes, notably changes in the behavioral determinants (defined according to the HAPA), plaque, and self-reported oral health behavior, using linear regression analysis. Results from all analyses will be presented with the 95% CI. For all analyses, the SEP stratum will be used as the primary covariable, while sociodemographic characteristics for which a baseline difference is observed will be used as secondary covariables. Such postrandomization differences between groups will be explored using descriptive statistics. A difference of 20% for discrete variables, a full standard deviation (pooled) for normally distributed data, and a quartile for nonnormally distributed data will be used as thresholds for similarity.

### Cost-Effectiveness

#### Measurement and Inventory of Costs

The *Dutch Manual for Costing Studies in Health Care* will guide the identification of costs [49]. Cost prices will be calculated individually, which means that the frequency and reason for visits to the dental clinic will be requested for both groups. Based on this information, a cost price can be calculated. For the intervention group, the cost for the add-on TOHI will be based on the reported time that the OHC spent at each appointment.

#### Cost-Effectiveness Analysis

If the TOHI is shown to be superior as an additional treatment to usual care in the analysis of the principal outcome (using the imputed data set), we will analyze the balance between the effect and cost of the TOHI. We will then report the results of a cost-effectiveness analysis and a cost-utility analysis. Incremental cost-effectiveness ratios will be calculated by dividing the difference in total costs between conditions by the difference in average effect size. Because we will calculate costs over 48 months, correction for inflation will be considered. Bias-corrected and accelerated bootstrapping with 5000 replications will be used to calculate the 95% CI around the mean difference in total costs between the treatment groups. Bootstrapping will also be used to estimate the uncertainty surrounding the incremental cost-effective ratio, which will be graphically presented on cost-effectiveness planes. Results from the economic evaluation will be reported in line with the Consolidated Health Economic Evaluation Reporting Standards Statement [50].

## Results

The first parent-child dyads were enrolled in June 2017, and recruitment finished in June 2019. In total, 402 dyads have been randomized. Interventions and data collection for all of these dyads will be completed by the end of 2022. Therefore, final results are expected in the first half of 2023.

## Discussion

This paper describes the study protocol for a multisite, pragmatic RCT that will examine the effectiveness of an innovative approach in preventing ECC in Dutch preschool-age children. The literature on prevention strategies for ECC assumes that interventions initiated during pregnancy or in the first year of life have a good chance of success [9,51,52]. However, there is currently no consensus on targeting preschool-age children from the first year of life and effectively offering preventive care from first tooth eruption [16,53]. As a result, there is a gap in the current provision of preventive oral care, and knowledge is lacking among the target group and health professionals [15,54]. Furthermore, our study population is at risk of caries at an age at which oral health care is shown to be of low priority for many, even though health insurance fully covers it [8]. Therefore, the TOHI study has been welcomed by oral health professionals and public health care workers. With this pragmatic multicenter study, we aim to contribute to the current evidence base on preventive oral health care, particularly the effects of ECC preventive interventions in preschool children, and inform current practices and standards for dental care and youth health care.

Before initiating our TOHI study, 5 RCTs with similar objectives and populations in a public health setting had been published [21,55-59]. Since then, the results of another 8 RCTs have been published (as of February 2021) [21,51,52,60-65]. Comparing our study with these previous studies reveals similarities and differences. Most of these studies concerned oral health outcomes and the organization of care similar to ours to ensure early access to oral health promotion or dental care; most studies focused on target groups from the lower social classes. All studies started within the first year of the child’s life (or during pregnancy) and had caries incidence or cumulative caries incidence as the principal outcome measure. Interventions included repeated oral health messaging, handing out pamphlets or oral hygiene products, and providing maternal counselling using MI and anticipatory guidance, sometimes combined with professional fluoride applications. Only 1 of the published studies described a tailored intervention where counselling was based on an individual caries risk approach, such as was used in the TOHI [58]. To our knowledge, none of the published studies assigned oral health professionals as OHCs to WBCs to provide preventive care combined with the regular WBC visits. While some studies included nonrandomized comparisons or even used historical control groups, others used clustered randomization. The study sizes ranged from 187 to 1441. Follow up for study endpoints in the published studies ranged from 12 to 48 months, with some continuing to observe the children until school age. None of these studies continued the intervention after the age of 42 months.

When interpreting the results of this study, the strengths and weaknesses of the study design and conduct must be taken into account. First, a strength is that the TOHI is based on strategies that have proven to be successful in previous studies. We have interviewed parents, oral health professionals, and public health workers on their needs and priorities for promoting the oral health of preschool children. Representatives of these groups have contributed to the study design phase and regularly convened during the conduct of the study. The intervention principles have been translated into a standardized, structured approach with practical instructions for oral health coaches and parents, which has been tested in a small-scale feasibility study. Nevertheless, combining oral health promotion by OHCs at WBCs requires organizational changes in the provision of public and oral health care and commitment from parents.

Second, our study methods endeavored to prevent bias wherever possible by publishing the study design and transparently reporting the methods and procedures used for the determination of statistically appropriate sample size, the randomized, stratified, and concealed intervention allocation, and for blinding of study staff involved in collecting study data and analyzing study outcomes. However, our study was designed for real-life practice and required compromises. In particular, decisions about trade-offs between the optimum research design and the practicalities of delivering health care in the real world were challenging [66]. While the usual-care control condition (the current nationwide, municipal Public Health Service protocol at WBCs) was assumed to include recommendations and advice on oral health, a previous study has shown that little can be expected from this [15]. Therefore, the TOHI was studied as a pragmatic RCT and delivered as an add-on to usual care. A placebo for the TOHI to blind participants to the allocated add-on intervention was not feasible. Hence, information bias cannot be ruled out as influencing the study results because of the pragmatic study design.

Third, it is generally known that RCTs usually include participants with a higher level of education or greater motivation and interest. In anticipation of this, WBCs were selected after consultation with the municipalities to represent neighborhoods with a predominantly low-SEP population. In addition, during participant recruitment, much attention was paid to translating the patient information required by the medical ethics committee, being physically present at the WBCs, designing banners and flyers with accessible information, and providing information by telephone to those interested.

Finally, this study had a relatively long follow-up time compared to most other studies on this topic. Therefore, to ensure sufficient statistical power to detect clinical relevance and statistical significance for the principal endpoint, a 30% dropout rate was taken into account when estimating the initial sample size before the start of the intervention.

Dissemination and implementation of our TOHI approach are planned on different levels and in various directions. The dissemination plans include activities both scientific and practical, and therefore include activities such as publication of the results of the RCT in scientific journals and presentations at conferences, as well as activities in support of national oral health policy and practice. For the dissemination of knowledge to practice, distinctions can be made between dissemination plans on the micro, meso, and macro levels. Activities at the micro level include training courses, education and post–initial education, and online or offline workshops to update oral health professionals with knowledge about preventive oral care from first tooth eruption and provide practical tools. At the meso level, the project group investigated what was needed for sustainable implementation of the TOHI; this will be translated into ready-to-use implementation plans for the practices. At the macro level, the knowledge and results will be shared with local and national politicians, policymakers, and health insurers to examine how oral care for the very youngest individuals can be organized differently and how this should be financed. Finally, it should be noted that we have created a practice network in which, in terms of implementation and maintenance of change of practice, establishing ownership of the TOHI is our main goal.

## Appendix

Multimedia Appendix 1 Intervention description of the Toddler Oral Health Intervention following TIDier.

Multimedia Appendix 2 Example of patientrecord.

Multimedia Appendix 3 Example of oral health report.

Multimedia Appendix 4 Peer-reviewer report 1 from the Taskforce for Applied Research SIA (The Netherlands).

## Abbreviations

dmfs/dmft: decayed, missing, and filled surfaces and teeth
ECC: early childhood caries
HAPA: Health Action Process Approach
HLS-EU-Q16: 16-item European Health Literacy Survey Questionnaire
ICDAS: International Caries Detection and Assessment System
MI: motivational interviewing
MRC: Medical Research Council
NOCTP: Non-Operative Caries Treatment And Prevention
OHC: oral health coach
PUFA: visible pulpal involvement, ulceration caused by dislocated tooth fragments, fistula, and abscess
RCT: randomized controlled trial
SEP: socioeconomic position
TIDieR: Template for Intervention Description and Replication
TOHI: Toddler Oral Health Intervention
WBC: well-baby clinic
