# Supplementary material for: Oral Health Coaches at Well-Baby Clinics to Promote Oral Health in Preschool Children From the First Erupted Tooth: Protocol for a Multisite, Pragmatic Randomized Controlled Trial
Source: JMIR Res Protoc. 2022 Aug 31;11(8):e39683. doi: 10.2196/39683 (PMC9475409; doi:10.2196/39683)
Supplement: Multimedia Appendix 1 [file resprot_v11i8e39683_app1.docx]

## Multimedia Appendix 1. Intervention description of TOHI following TIDier

|  | **Reach children at well-baby clinics** | **Non-Operative Caries treatment and prevention method** | **Motivational interviewing** | **The Health Action Process Aproach** |
| --- | --- | --- | --- | --- |
| **Why**  Describe any rationale, theory or goal of the elements essential to the intervention | In the Toddler Oral Health Intervention (TOHI), an oral health coach (OHC) is seconded from a dental clinic to a participating WBC in the same neighbourhood. Reaching very young children and their parents through the WBCs has proven successful in the nationwide Scottish Childsmile programme [17,18].  Oral health care professionals experience difficulties reaching out to children below age five. Less than half of the children had at least one dental visit at the age of four [12,13]. At the same time, more than 90% of all newborns, including children from ethnic minority groups and low socioeconomic backgrounds, visit well-baby clinics (WBCs) for preventive health care and vaccinations at regular intervals up to four [14]. Therefore, WBCs appear to provide a unique window of opportunity for early oral health promotion. | The OHC works according to the Non-Operative Caries Treatment and Prevention (NOCTP) approach, which has been reported to result in a 40% reduction in caries incidence in Dutch schoolchildren [19]. The protocol has been adapted and applied to 0-4 years old (figure 3). The general understanding underlying NOCTP is that caries is a localized process that (to a large extent) can be prevented by toothbrushing with fluoride toothpaste and low sugar intake frequency. With this understanding in mind, the OHC aims to create awareness among parents that through establishing adequate oral health behavior, they can largely contribute to preventing caries for their child. The standardized protocol within the NOCTP method contributes to an uniform and transparent approach for OHC and parents. | While motivational interviewing (MI) was initially developed as a behavioral technique for treating substance abuse [20], it has been shown to be effective in contributing to the prevention of ECC: ranging from a 16-26% reduction in caries incidence and a 30-55% reduction in the number of decayed teeth (21-24). Motivational interviewing (MI) is used in TOHI to elicit parents’ internal motivation and explore barriers and facilitators for desired oral health behavior. | The Health Action Process Approach (HAPA) [25], a theoretical framework to explain, predict, and modify health behaviors, provides tools to focus on the underlying determinants of behavior. By understanding the stages of behavioral change and focusing the intervention on the associated determinants, the impact of NOCTP and MI could be increased. The importance of determinants that underpin motivational and self-regulatory processes and help translate intention into behavior in oral health care, such as attitudes, self-efficacy, planning, and action control, has been demonstrated in previous research [26-29]. |
| **What**  Materials: Describe any physical or informational materials used in the intervention, including those provided to patients or used in intervention delivery or in the training of intervention providers. | All children receive the usual standard care during treatment. Children in the intervention group receive TOHI in addition. In combination with the regular WBC appointments, parent-child dyads have an appointment with the OHC from the first tooth coming through around 6-11 months. Materials used by the OHC are:   - A penlight; - Disposables (mouth mirror, cotton wool rolls, napkins, toothpicks, gloves, mouth masks, alcohol wipes); - A oral hygiene demonstration puppet; - Tooth health booklet for parents; - Free toothbrush and toothpaste; - Reward stickers. | All OHC receive a one- day training in the NOCTP method offerd by the Dutch Oral Health association (Dutch translation: Ivoren Kruis). Patient records developed for TOHI have a NOCTP diagram showing caries risk and recall interval. For parents, the caries risk of their child is translated in the oral health reports. | All OHC receive training from an experienced MI trainer specialized in training oral health professionals. The training comprises the theory of MI and stages of change, practice, role-plays, peer feedback and professional feedback on audiotaped interventions.  The first training starts before the start of the project when participants are enrolled. Training will be continued during the project with an interval of 3-4 months. | All OHCs are trained in the use of HAPA theory. In addition, printed patient records will be used by the OHC. This record guides them through the process (appendix 2).  Essential aspects of the HAPA theory are included in the oral health reports of the healthy-toddler-teeth booklet for parents (appendix 3). |
| **What**  Procedures: Describe each of the procedures, activities, and/or processes used in the intervention, including any enabling or support activities. | All participants enrolled in the intervention group will be approached by the OHC between 6 and 11 months. An appointment with the OHC will be combined with a scheduled appointment at the WBC. After the first appointment, all appointments will be planned in coordination with the WBC nurse and the OHC. | During all appointments with the OHC, an assessment will be made of the individual cariës risk of the child using the NOCTP methodology. Depending on the risk and the associated recall interval, the next appointment with the OHC will be combined with the next WBC appointment, or an additional appointment will be scheduled.  Depending on the caries risk, self-care agreements are made and reported in both the patiëntrecord as well in the oral health reports for parents. | MI techniques mainly used by the OHC are dealing with resistance, showing empathy, affirmation, reflection and exploring ambivalent feelings. | At each appointment, the OHC estimates the stage of behavioral change concerning oral hygiene behavior and diet. Based on the stage, the intervention target can be determined (i.e. an informed parent who is aware of risk perception, outcome expectations and self-efficacy will skip regularly brushing their child's teeth. As a result, the OHC can focus on action and coping planning rather than repeating information). |
| **Where**  Describe the location where the intervention occurred, including any necessary infrastructure. | All parent-child pairs are recruited into the WBC. The appointments with the OHC are combined with scheduled appointments in the WBC to minimise the burden on the parents. | | | |
| **When and How Much**  Describe the number of times the intervention was delivered and over what period of time, including the number of sessions, their schedule, and their duration, intensity, or dose. | After randomization, the first appointment (10-20 minutes) with the OHC will be scheduled at the age of 6 -11 months. All subsequent appointments will initially follow regular WBC visits at the age of 8-11, 15, 18, 24, 36 and 42 months. If necessary, the recall interval can be shortened based on caries risk. | | | |
| **Who**  For each category of intervention provider (such as a psychologist, nursing assistant), describe their expertise, background, and any specific training given. | Eligible OHCs for this study should have a background in dentistry (dentist, dental hygienist or dental assistant). They should also be prevention-minded, trained (or willing to be trained) in the NOCTP method, have good social communication skills, and experience paediatric dentistry. In addition, all OHCs receive training in the TOHI intervention delivered by a certified MI trainer and the principal investigator, both experienced in training oral health care professionals and students in NOCTP, HAPA and MI. A paediatric dietitian will provide extra training on infant and toddler nutrition related to oral health. The first training takes place before the study starts. Subsequently, training continues every 3-4 months for the duration of the study. During this training, the focus will be on recognizing stages of health behavior, using HAPA, and developing advanced MI skills such as recognizing and managing behavioral resistance and detecting ambivalence. Learning these skills takes time and is an ongoing process. Therefore, the training consists of practical examples and casuistics provided by the OHCs, role plays and peer feedback. In addition, the MI trainer provides individual feedback on self-recorded audio fragments that are uploaded by the OHCs. | | | |
| **How**  Describe the modes of delivery (such as face to face or by some other mechanism, such as internet or telephone) of the intervention and whether it was provided individually or in a group. | Interventions are individually and face to face. | | | |

stylefix
