# Supplementary material for: Oral Health Coaches at Well-Baby Clinics to Promote Oral Health in Preschool Children From the First Erupted Tooth: Protocol for a Multisite, Pragmatic Randomized Controlled Trial
Source: JMIR Res Protoc. 2022 Aug 31;11(8):e39683. doi: 10.2196/39683 (PMC9475409; doi:10.2196/39683)
Supplement: Multimedia Appendix 2 [file resprot_v11i8e39683_app2.pdf]

11 months consult

Personal ID: _______

Date: _____________

Total consultation time: _______minutes

Which teeth are present:

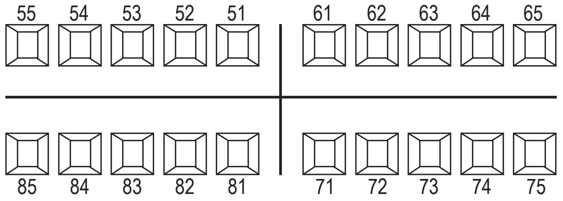

Age-related topics:

(Check what has been discussed)

- □ In-between eruption phases (first molars will erupt around 12-15 Months)
- □ Advice to brush twice daily with fluoride toothpaste
- □ How to brush the child's teeth (position child and parent)
- □ No comfort bottle feeding or bottle in bed (water is allowed anytime)
- □ Can the baby already drink from a cup? Offer training tips for practicing.
- □ Limit the frequency of sugarconsumption to max. 7 times a day (water is allowed anytime).
- □ Try to stop dummy sucking during daytime.

NOCTP caries risk assessment, oral health, oral hygiene and oral health behaviour (check what is applicable)

**Criterion**	**Assessment**	□ **Low risk**	□ **Increased risk**	□ **High risk**
Parental involvement?<br>*Measured by attitude towards oral health, oral health knowledge, childs' oral hygiene and adherence to general advices.for caries prevention.*	□ Sufficient (0 points)<br>□ Insufficient (1 point	0 points	1 point<br>(no cariesactivity)	1 or 2 points<br>(with cariesactivity)
Is there any active enamel- or dentin caries?	□ No (0 points)<br>□ Yes (1 point)<br>*(mark element and surface in diagram)*<br><br>Total points: _______	Action:<br>• Oral examination<br>• Complete oral report in healthy-toddler-teeth booklet (optional: formulate action plan)<br>• Discuss relevant age-related topics	Action:<br>• Oral examination.<br>• Complete oral report in healthy-toddler-teeth booklet + action plan.<br>• Discuss relevant age-related topics.	Action:<br>• Oral examination.<br>• Complete oral report in healthy-toddler-teeth booklet + action plan.<br>• Discuss relevant age-related topics.

Dental plaque:
- □ None
- □ Local
- □ Generalised

Gingiva:
- □ Healthy
- □ Local gingivitis
- □ Generalised gingivitis

Any remarks about soft tissues:
If yes, ________________
____

Feeding at night:
- □ No
- □ Yes, bottle feeding
- □ Yes, breastfeeding

Parafunctions:
- □ No
- □ Tongue pressing
- □ Lisping
- □ Infantile swallow
- □ Thumb/finger sucking
- □ Dummy sucking

Toothbrushing:
- □ 1 time a day
- □ 2 times a day
- □ Other: ____________

Toothbrush:
- □ Manual toothbrush
- □ Elect. toothbrush
- □ Varying

Toothpaste
- □ <1000 ppm F toothpaste
- □ 1350-1500 ppm F toothpaste
- □ Other: ______________

Remarks about oral hygiene:
____
____
____

Stage of oral health behaviour change and behavioural determinants for focus coaching (check what is applicable):

□ Pre-contemplation<br>□ Contemplation<br>□ Relapse	□ Preparation<br>□ Action	□ Maintainance
Intervention goal:<br>Intention forming	Intervention goal:<br>Planning behaviour	Intervention goal:<br>Maintain behaviour
Focus on determinants:<br>□ Self-efficacy<br>□ Outcome expectancies<br>□ Risk Perception	Focus on determinants:<br>□ Action self-efficacy<br>□ Action Planning<br>□ Coping Planning	Focus on determinants:<br>□ Recovery self-efficacy<br>□ Action control

Explanatory note: ______________________
____
____
____

Is this behavioural change phase also valid for the attitude towards food (e.g. drinking water?) □ Yes □ No

Explanatory note: ______________________
____
____
____

Advice given and/or agreed action plans: ______________________
____
____
____

Evaluate next time: ______________________
____
____
____
____

Next appointment:

- □ with ___ months consult at WBC
- □ extra appointment in ___month(s)

Details next appointment:

- □ Monday
- □ Tuesday
- □ Wednesday
- □ Thursday
- □ Friday

Date: ___/___/______

Time: ___:___
