# Supplementary material for: Oral Health Coaches at Well-Baby Clinics to Promote Oral Health in Preschool Children From the First Erupted Tooth: Protocol for a Multisite, Pragmatic Randomized Controlled Trial
Source: JMIR Res Protoc. 2022 Aug 31;11(8):e39683. doi: 10.2196/39683 (PMC9475409; doi:10.2196/39683)
Supplement: Multimedia Appendix 3 [file resprot_v11i8e39683_app3.pdf]

11 Months

Oral Health Report

Date:

Which teeth do I already have?

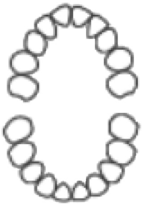

Total:

Remarks about teeth:

How healthy are my teeth?

My teeth are brushed ... times a day	✔	✖
My teeth are clean	✔	✖
I have ... (incipient) cavities	✔	✖
I eat or drink ... times a day	✔	✖

We received compliments for:

We must pay extra attention to:

Oral health action plan

What we will do:

When will we do this:

A potential pitfall here could be:

What is our copingstrategy for this pitfall:

How important do we find this to succeed:

1 2 3 4 5 6 7 8 9 10

Not important at all — Very important

Our confidence that it will succeed?

1 2 3 4 5 6 7 8 9 10

Not confident at all — Very confident

New appointment:

- ☐ Together with WBC appointment at ... months
- ☐ Extra appointment in ... weeks/months

Details new appointment:

Day:	Date:	Time:
Mon / Tue / Wed / Thur / Fri		
