# Supplementary material for: Oral Health Coaches at Well-Baby Clinics to Promote Oral Health in Preschool Children From the First Erupted Tooth: Protocol for a Multisite, Pragmatic Randomized Controlled Trial
Source: JMIR Res Protoc. 2022 Aug 31;11(8):e39683. doi: 10.2196/39683 (PMC9475409; doi:10.2196/39683)
Supplement: Multimedia Appendix 4 [file resprot_v11i8e39683_app4.pdf]

SiA

Aan het College van Bestuur
Hogeschool Utrecht
T.a.v. dhr. J. Bogerd
Postbus 573
3500 AN UTRECHT

Datum:	15-12-2016
Kenmerk:	SVB / RAAK.PUB03.018
Onderwerp:	Besluit inzake aanvraag subsidieregeling RAAK-publiek voor het project:
Projectnaam:	Gezonde Peutermonden - de mondzorgcoach als JGZ innovatie
Dossiernummer:	RAAK.PUB03.018
Budgetnummer:	2233

Geachte heer Bogerd,

Op 28 juni 2016 heeft het Nationaal Regieorgaan Praktijkgericht Onderzoek SIA (Regieorgaan SIA), onderdeel van NWO, uw aanvraag met bijbehorend voorstel voor het project *Gezonde Peutermonden - de mondzorgcoach als JGZ innovatie* ontvangen. Met deze aanvraag wenst u in aanmerking te komen voor verstrekking van subsidie in het kader van de regeling RAAK-publiek (call 2016).

Tot ons genoegen kunnen wij u berichten dat uw aanvraag is gehonoreerd.

Procedure
Aanvragen worden getoetst op volledigheid. Aanvragen die aan alle formele vereisten voldoen, worden aan de beoordelingscommissie voorgelegd. De beoordelingscommissie beoordeelt de projecten op basis van de beoordelingscriteria zoals deze zijn vastgelegd in de regeling RAAK-publiek (call 2016), te weten vraagarticulatie, netwerkvorming en onderzoeksplan. Uitsluitend projectvoorstellen die op deze drie beoordelingscriteria voldoende scoren, worden door de beoordelingscommissie van positief advies voorzien. In het kader van de beoordeling wordt de hogeschool in de gelegenheid gesteld schriftelijk te reageren ('hoor en wederhoor'). De beoordelingscommissie brengt advies uit aan het bestuur van Regieorgaan SIA. Het bestuur van Regieorgaan SIA toetst of de gevolgde procedure op de juiste wijze is doorlopen en besluit op basis van het advies van de beoordelingscommissie.

Beoordeling
In deze call zijn 62 aanvragen met bijbehorende projectvoorstellen ingediend. Hiervan komen 24 projectvoorstellen voor subsidie in aanmerking.
Naar het oordeel van de beoordelingscommissie scoort uw projectvoorstel als volgt:

- Vraagarticulatie: 4,60
- Netwerkvorming: 4,80
- Onderzoeksplan: 4,80

Nationaal Regieorgaan Praktijkgericht Onderzoek SIA
Van Vollenhovenlaan 661 • Postbus 3021 • 3502 GA Utrecht • 030 - 600 13 80 • www.regieorgaan-sia.nl

Het Nationaal Regieorgaan Praktijkgericht Onderzoek SIA is onderdeel van de Nederlandse Organisatie voor Wetenschappelijk Onderzoek (NWO)

SiA

Uw totaalscore bedraagt 4,75 en is opgenomen op plaats 8 in de ranking van de 24 projectvoorstellen die voor subsidie in aanmerking komen.

De beoordeling wordt als volgt toegelicht:
Het proces van vraagarticulatie vindt de commissie voldoende. De commissie herkent een maatschappelijk relevant vraagstuk die aansluit bij de kennisvragen van professionals. Het proces is echter vrij beperkt beschreven, waardoor het de commissie onduidelijk is hoe de jeugdgezondheidszorg is bevraagd om tot de geformuleerde praktijkvraag te komen.
Het beoogde netwerk is volgens de commissie ruim voldoende. Het consortium bestaat uit een sterk netwerk van zowel kennisinstellingen en praktijkpartners. Eerdere samenwerking tussen de partners geeft vertrouwen voor de duurzaamheid van het netwerk. De commissie mist echter expliciete betrokkenheid van jeugdartsen en verpleegkundigen en betrokkenheid van de einddoelgroep (ouders met kinderen).
De commissie beoordeelt de kwaliteit van het onderzoeksplan als ruim voldoende. De state-of-the-art is helder beschreven en het onderzoeksplan bevat een duidelijke vraagstelling en onderbouwing van de gekozen methodiek. De commissie heeft veel waardering voor de combinatie van RCT met kwalitatief onderzoek. De kwalitatieve onderzoeksmethode zou echter nog verder toegelicht kunnen worden (beschrijving van focusgroep en interviews) en de commissie vraagt aandacht voor de deelname van ouders en kinderen met lage SES.

De aanvrager heeft op bovenstaande opmerkingen gereageerd middels een weerwoord.
De commissie waardeert de reactie van de aanvrager, waarin een adequate toelichting wordt gegeven op de opmerkingen van de commissie.

De beoordelingscommissie heeft geoordeeld dat uw projectvoorstel op alle criteria voldoende scoort. Dit eindoordeel van de beoordelingscommissie is voorgelegd aan het bestuur van Regieorgaan SIA. Het bestuur heeft vastgesteld dat de procedure op de juiste wijze is doorlopen en heeft op basis van dit eindoordeel en de goedgekeurde, ingediende begroting besloten dat uw projectvoorstel voor subsidie in aanmerking komt.

Subsidieontvanger
De penvoerende hogeschool geldt als subsidieontvanger. De contactgegevens zoals bekend bij Regieorgaan SIA, zijn als volgt:

Hogeschool Utrecht
Postbus 573
3500 AN UTRECHT

De contactpersoon voor de penvoerder is:
heer Soentken
menno.soentken@hu.nl
